# Integrating transcriptomics, glycomics and glycoproteomics to characterize hepatitis B virus-associated hepatocellular carcinoma

**DOI:** 10.1186/s12964-024-01569-y

**Published:** 2024-04-01

**Authors:** Zhuo Li, Na Zhang, Zewen Dong, Xin Wang, Jian Zhou, Juan Gao, Yunyun Yang, Jing Li, Feng Guan, Yue Zhou, Zengqi Tan

**Affiliations:** 1 Department of Laboratory Medicine, The First Affiliated Hospital of Xi’an Medical University, Xi’an, Shaanxi 710077 P.R. China; 2 Key Laboratory of Resource Biology and Biotechnology in Western China, Ministry of Education, Provincial Key Laboratory of Biotechnology, College of Life Sciences, Northwest University, Xi’an, Shaanxi 710069 P.R. China; 3 Institute of Hematology, Provincial Key Laboratory of Biotechnology, School of Medicine, Northwest University, Xi’an, Shaanxi 710069 P.R. China

**Keywords:** HBV-associated HCC, Transcriptomics, Glycomics, Glycoproteomics, Fucosylation

## Abstract

**Background:**

Hepatocellular carcinoma (HCC) ranks as the third most common cause of cancer related death globally, representing a substantial challenge to global healthcare systems. In China, the primary risk factor for HCC is the hepatitis B virus (HBV). Aberrant serum glycoconjugate levels have long been linked to the progression of HBV-associated HCC (HBV-HCC). Nevertheless, few study systematically explored the dysregulation of glycoconjugates in the progression of HBV-associated HCC and their potency as the diagnostic and prognostic biomarker.

**Methods:**

An integrated strategy that combined transcriptomics, glycomics, and glycoproteomics was employed to comprehensively investigate the dynamic alterations in glyco-genes, N-glycans, and glycoproteins in the progression of HBV- HCC.

**Results:**

Bioinformatic analysis of Gene Expression Omnibus (GEO) datasets uncovered dysregulation of fucosyltransferases (FUTs) in liver tissues from HCC patients compared to adjacent tissues. Glycomic analysis indicated an elevated level of fucosylated N-glycans, especially a progressive increase in fucosylation levels on IgA1 and IgG2 determined by glycoproteomic analysis.

**Conclusions:**

The findings indicate that the abnormal fucosylation plays a pivotal role in the progression of HBV-HCC. Systematic and integrative multi-omic analysis is anticipated to facilitate the discovery of aberrant glycoconjugates in tumor progression.

**Supplementary Information:**

The online version contains supplementary material available at 10.1186/s12964-024-01569-y.

## Introduction

Hepatocellular carcinoma (HCC) is a primary liver malignancy and ranks as the third leading cause of cancer-related death worldwide [1]. Hepatitis B and C viral infections are the two major etiologies of HCC [2, 3], with chronic HBV being predominating in China due to the high prevalence of HBV infection [4, 5]. The relative risk of HCC in HBV-positive patients compared to HBV-negative controls is much higher [6]. Most HBV-HCC patients experience a trilogy of hepatitis-cirrhosis-HCC [7, 8]. Alpha-fetoprotein (AFP) is the most commonly used serum biomarker for HCC since it was discovered in 1964 [9]. However, approximately 30% of patients with early-stage HCC are AFP negative [10], and elevated AFP levels are also detected in patients with cirrhosis or chronic HCV exacerbations [11]. Indeed, most cancer markers used in clinics are glycoproteins [12], and their glycan moieties may be structurally different in cancer [13]. Therefore, a global analysis of the precise variation of glycoconjugates in HBV- HCC is urgently needed.

Glycosylation is one of the most abundant and diverse protein post-translational modifications in eukaryotes, with profound effects on protein function, stability, subcellular localization and other traits [14]. N-glycosylation, the main form of protein glycosylation, was dysregulated in common diseases such as cancer [15–17], inflammation [18], Alzheimer’s disease [19, 20] and diabetes [21–23]. Connor A West et al. reported that the levels of tetra-antennary and fucosylation was elevated in HCC tissues compared to either cirrhotic tissue or adjacent untransformed tissue [24]. N-glycans with β1,6-N-acetylglucosamine (β1,6-GlcNAc) branching and sialylated GlcNAc structures were increased in HCC cell line, Huh7 [25].

As one of the terminal modifications, fucosylation is the process of transferring fucose from GDP-fucose to their substrates by fucosyltransferases in all mammalian cells [26]. Recent studies revealed that levels of core and outer-armer fucosylation were increased in the serum of patients with HCC [27]. The cancer-promoting capacity of cancer-associated fibroblasts was facilitated by modifying EGFR core fucosylation in non-small cell lung cancer [28]. Some highly abundant serum proteins such as transferrin (TF) and alpha-1-anti-trypsin (A1AT) were found to be decorated with increased levels of fucosylation in HCC [29]. These results demonstrate that glycosylation shows the potential as an indicator of liver disease progression to HCC. However, these studies commonly focus on cancer and adjacent tissues, rather than the process of liver disease from healthy control to HCC. In addition, each omics approach, such as transcriptomics, glycomics and glycoproteomics, cannot capture the entire biological complexity of most human diseases. Therefore, an integration of multiple omics approaches can provide a more comprehensive information on biological significance and diagnostic potential of glycans in the process of HCC.

In this study, multi-omics including transcriptomics, glycomics, and glycoproteomics were integrated to profile the aberrant levels of glycan-related gene, glycan pattern, site-specific glycoproteins in HBV-HCC process.

## Materials and methods

### Patients and samples

Three pairs of HBV-HCC and adjacent tissues, and serum samples from 27 chronic hepatitis B (CHB), 22 HBV-related liver cirrhosis (LC), 31 HBV- HCC patients and 20 healthy controls (HC) were collected from The First Affiliated Hospital of Xi’an Medical University. Informed consent was conducted in accordance with Declaration of Helsinki and experiments were approved by The First Affiliated Hospital of Xi’an Medical University Ethics Committee. Characteristic of samples are listed in Table S1&2.

### GEO data analysis

The gene expression profile data GSE135631 and GSE94660 was downloaded from GEO of the National Center for Biotechnology Information (NCBI). Gene expression data deposited as the Series Matrix data was used for analysis of the fold change of gene expression and *p* value.

### Quantitative reverse transcription PCR (RT-qPCR)

RNA from the indicated tissues was extracted using Trizol (ABclonal; Wuhan, Hubei, China) and reversed transcribed using the cDNA Reverse Transcription Kit (ABclonal). RT-qPCR was performed using SYBR Green Master Mix using Real-Time PCR system (TIANLONG; Xi’an, Shaanxi, China). Primer sequences were provided in Table S3.

### Analysis of N-glycans

Glycomic analysis was performed as previously described [30]. A volume of 10 μL serum was added onto a size-exclusion spin ultrafiltration unit (Millipore; Billerica, MA, USA). Proteins were denatured with 8 M urea/ 50 mM NH_4_HCO_3_, reduced with dithiothreitol (DTT), alkylated with iodoacetamide (IAM), and digested with PNGase F overnight at 37 °C. Released N-glycans were collected by centrifugation, lyophilized, and desalted using HyperSep Hypercarb solid phase extraction (SPE) cartridge (ThermoFisher Scientific; Waltham, Massachusetts, US).

Lyophilized N-glycans were dissolved and spotted onto an MTP AnchorChip sample target with 20 mg/mL 2,5-dihydroxybenzoic acid (DHB) as the matrix. Measurements were taken in positive-ion mode. Each full-mass scan was performed with following conditions: acceleration voltage, 24.59 kV; reflector voltage, 26.6 kV; pulsed ion extraction, 100 ns; mass range, 1000–3500 m*/z*. For the data analysis, the peaks were smoothed and baseline subtraction performed three times. Relative intensity was analyzed and generated using FlexAnalysis software (Bruker Daltonics; Bremen, Germany) based on MALDI-TOF/TOF–MS intensity. *m/z* data were obtained and annotated using GlycoWorkbench software program [31], cross-referenced with previous studies [32–34], together with knowledge of mammalian glycan biosynthetic pathway products. For the isomeric structures that are not easily deciphered by MALDI-TOF–MS, uncertainties in sequences, especially fuzzily defined capping unit locations, have been indicated outside the bracket in the proposed N-glycan structure.

### Proteomics and glycoproteomics

Protein extraction and digestion was performed as previously described [30]. In brief, the serum protein was denatured in 8 M urea/ 50 mM NH_4_HCO_3_, then reduced with DTT, alkylated with IAM, digested with lysyl endopeptidase (Promega; Madison, WI, USA) for 4 h at 37 °C, and then incubated with trypsin (Promega) overnight at 37 °C with shaking. The digested peptides were acidified with 10% trifluoroacetic acid to pH < 3, collected by centrifugation and purified using Oasis HLB cartridges (Waters; Milford, MA, USA). Desalted peptides were subjected to LC–MS/MS analysis for proteomics. N-glycopeptides were enriched by MAX column (Waters) using desalted peptides, and subjected to LC–MS/MS analysis for glycoproteomics as described previously [35].

### Enzyme-Linked Immunosorbent Assay (ELISA)

To determine the Igs levels in serum, direct ELISA was applied. ELISA plates were coated with serum sample for 2 h at 37 °C, blocked with 3% BSA in PBS for 2 h at room temperature (RT), rinsed with PBST, incubated with antibody against IgA1 or IgG2 (Abcam; Cambridge, MA, USA) for 2 h at 37 °C, rinsed with PBST, incubated with secondary antibody for 30 min at RT, rinsed with PBST, and visualized with TMB substrate kit (Beyotime Institute of Biotechonology; Haimen, China). The absorbance values were determined at 450 nm with a plate reader.

To determine the glycosylation on Igs, sandwich ELISA was performed. ELISA plates were coated with antibodies against IgA1 or IgG2 overnight at 4 °C, rinsed with PBST, blocked with 5% BSA, incubated with serum samples for 2 h at 37 °C, rinsed with PBST, incubated with biotinylated lectins (Vector Labs) overnight at 4 °C, incubated with VECTASTAIN ABC reagent for 30 min at RT, and visualized with TMB substrate solution. The absorbance values were determined at 450 nm with a plate reader.

### Data analysis

All experiments were reproduced at least three times. All values were presented as mean ± standard deviation (SD). Two-tailed Student’s t-test was used for comparison of data sets between two groups and differences with *p* < 0.05 were considered statistically significant. Statistical analysis was performed using GraphPad Prism 9.3.1 software program.

## Results

### Glycan-related genes expression of HBV-HCC and adjacent tissues

**Fig. 1:**
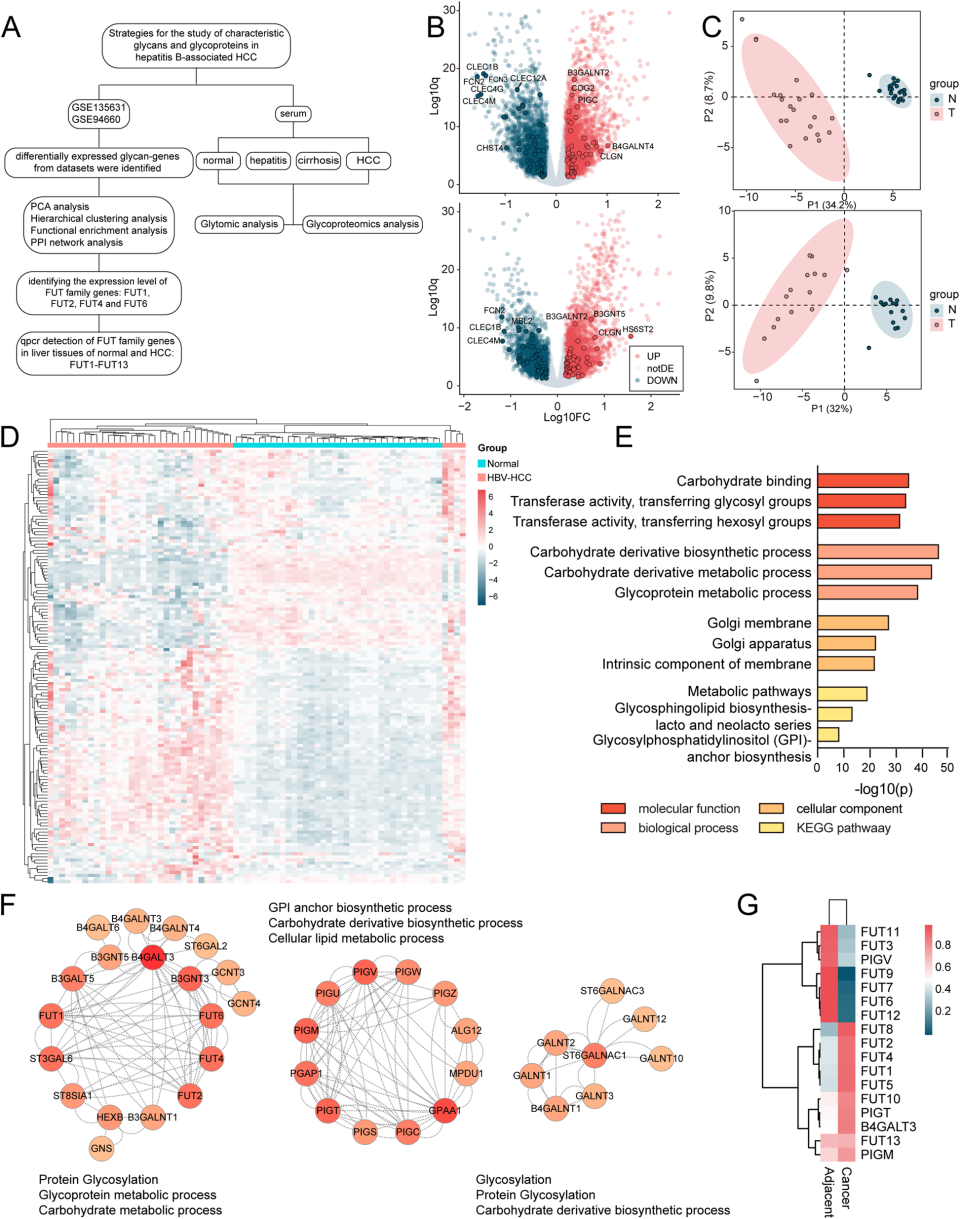
Differentially expressed glycan-related genes in HBV-related HCC of GSE135631 and GSE94660. **A** Workflow of the present study. **B** Volcano plot of expression patterns of identified genes in GSE135631 and GSE94660. Red dots: up-regulated genes. Green dots: down-regulated genes. Highlighted dots: DEGGs. The q value (log10) is plotted against the log10 (FC: HBV-HCC tissues vs. adjacent tissues) using the cut-offs of fold change > 1.5 or < 0.67 and *p* value < 0.05. **C** PLS-DA plot of DEGGs in GSE135631 and GSE94660. **D** Heatmap showing the expression pattern of DEGGs in HBV-HCC and adjacent tissues of GSE135631 and GSE94660. **E** Functional enrichment analysis of DEGGs. **F** The PPI network of DEGGs performed by the STRING database and cytoscape tools. The red colour intensity was proportional to the degree of connectivity. **G** The mRNA expression of 13 FUTs, PIGV, PIGT, PIGM and B4GALT3 genes in 3 pairs of HBV-HCC and adjacent tissues determined by RT-qPCR

A better understanding of glyco-gene expression changes may facilitate the exploration of association between glycosylation and HBV-HCC. The differences in glyco-gene expression and glycosylation in HBV-HCC were investigated using an integrated strategy with a combination of transcriptomics, glycomics and glycoproteomics (Fig. 1A). First, GSE135631 and GSE94660 gene expression profiles, including 15 and 21 pairs of HBV-HCC and adjacent tissues respectively, were retrieved from the GEO database. A total of 7502 and 7839 genes were differentially expressed in GSE135631 and GSE94660, as shown in Fig. 1B, in which top 100 differentially expressed glyco-genes (DEGGs) were highlighted (Fig. 1B, Table S4). Partial Least Squares Discrimination Analysis (PLS-DA) of these DEGGs exhibited a clear separation of HBV-HCC from adjacent tissues (Fig. 1C). The expression patterns of all DEGGs across HBV-HCC and adjacent tissues from the combined two GSE study were shown as a heatmap (Fig. 1D). Next, functional enrichment analysis revealed that the main molecular function categories were carbohydrate binding, transferring glycosyl and hexosyl group activity (Fig. 1E). Protein–protein interaction (PPI) network of these DEGGs was constructed in an attempt to explicit the molecular mechanisms in the progression of HBV-HCC. Clustering analysis revealed ten major modules, in which protein glycosylation, GPI anchor biosynthetic process and glycosylation processes were mostly enriched (Fig. 1F; S1). Hub genes with a high degree of connectivity in the PPI network are significantly enriched in the process of fucosylation (FUT4, FUT6, FUT2 and FUT1), GPI anchor biosynthesis (PIGM, PIGV, PIGT and GPAA1), galactosylation (B4GALT3) and galNAcylation (B3GNT3).

Furthermore, FUTs family expression at mRNA levels were determined by RT-qPCR in HBV-HCC and adjacent tissues (Fig. 1G), revealed an elevated level of FUT1, FUT2, FUT4, FUT5, FUT8, FUT10, PIGT, PIGM and B4GALT3, as well as a reduced level of FUT3, FUT6, FUT7, FUT9, FUT11, FUT12 and PIGV in HBV-HCC tissues. Although HCC and adjacent tissue samples in transcriptomic analysis can not represent the whole HCC progression, these data provide clues in understanding dysregulated glycosylation especially fucosylation in HBV-HCC.

### N-glycan profiles of normal, hepatitis, cirrhosis and HCC serum samples

**Fig. 2:**
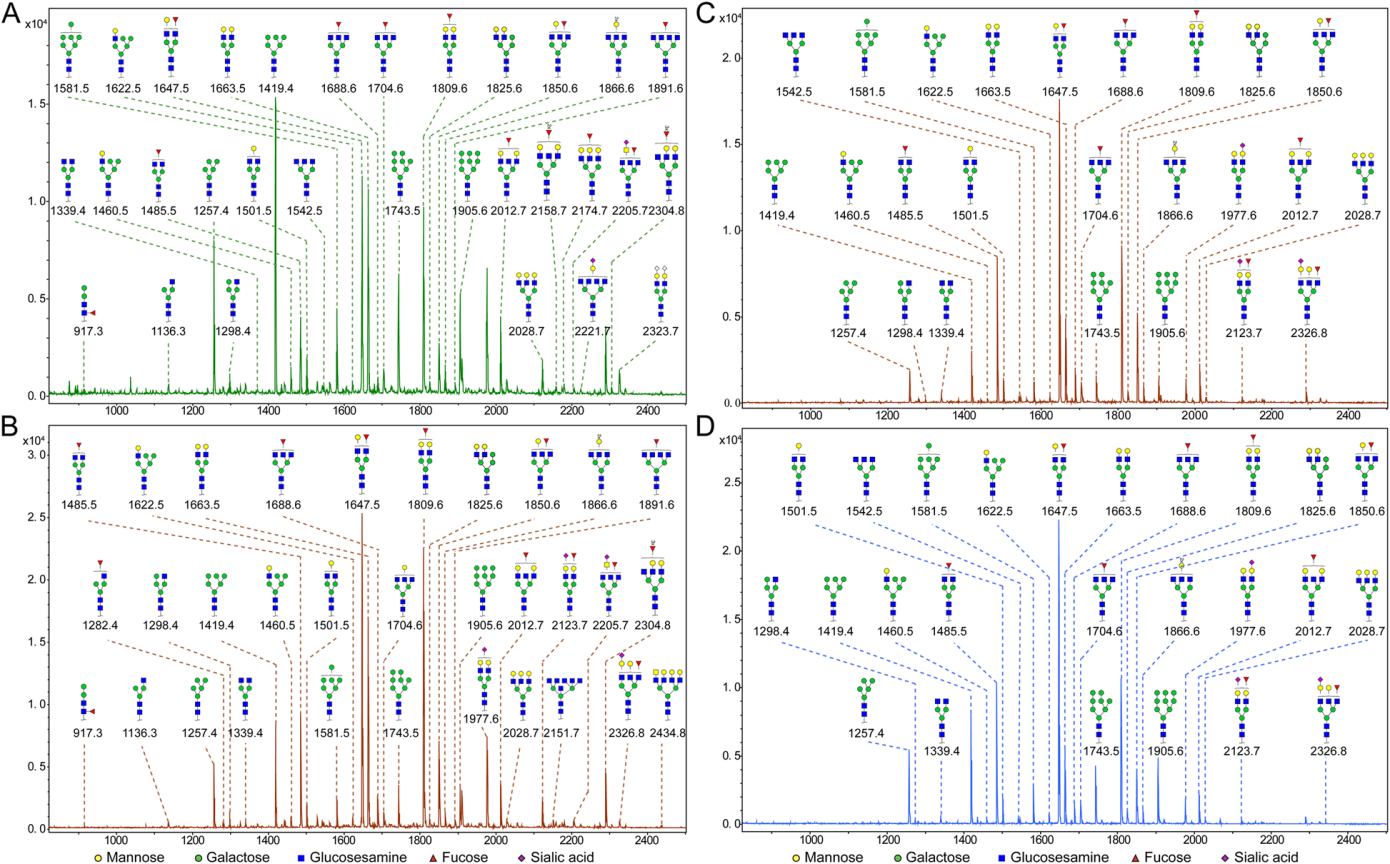
MALDI-TOF/TOF–MS spectra of N-glycans in HC, CHB, LC and HBV-HCC serum samples. Peaks of MALDI-TOF/TOF–MS spectra (signal-to-noise ratio > 5) were selected for relative intensity analysis in HC (**A**), CHB (**B**), LC (**C**) and HBV-HCC (**D**) samples. Detailed structures were annotated with GlycoWorkbench software. Proposed structures are indicated by *m*/*z* value

In our studies, N-glycans in HC, CHB, LC and HBV-HCC serum samples (Tab. S1) were profiled by MALDI-TOF/TOF–MS to reveal abnormal N-glycosylation. Representative MS spectra of N-glycans with signal-to-noise ratios > 5 were displayed and annotated (Fig. 2; Table S5). A total of 36 distinct *m/z* N-glycan structures were identified, with 31, 32, 25 and 25 N-glycans present in HC, CHB, LC and HBV-HCC samples, respectively. There were 21 N-glycans found in all groups but with different intensities.

**Fig. 3:**
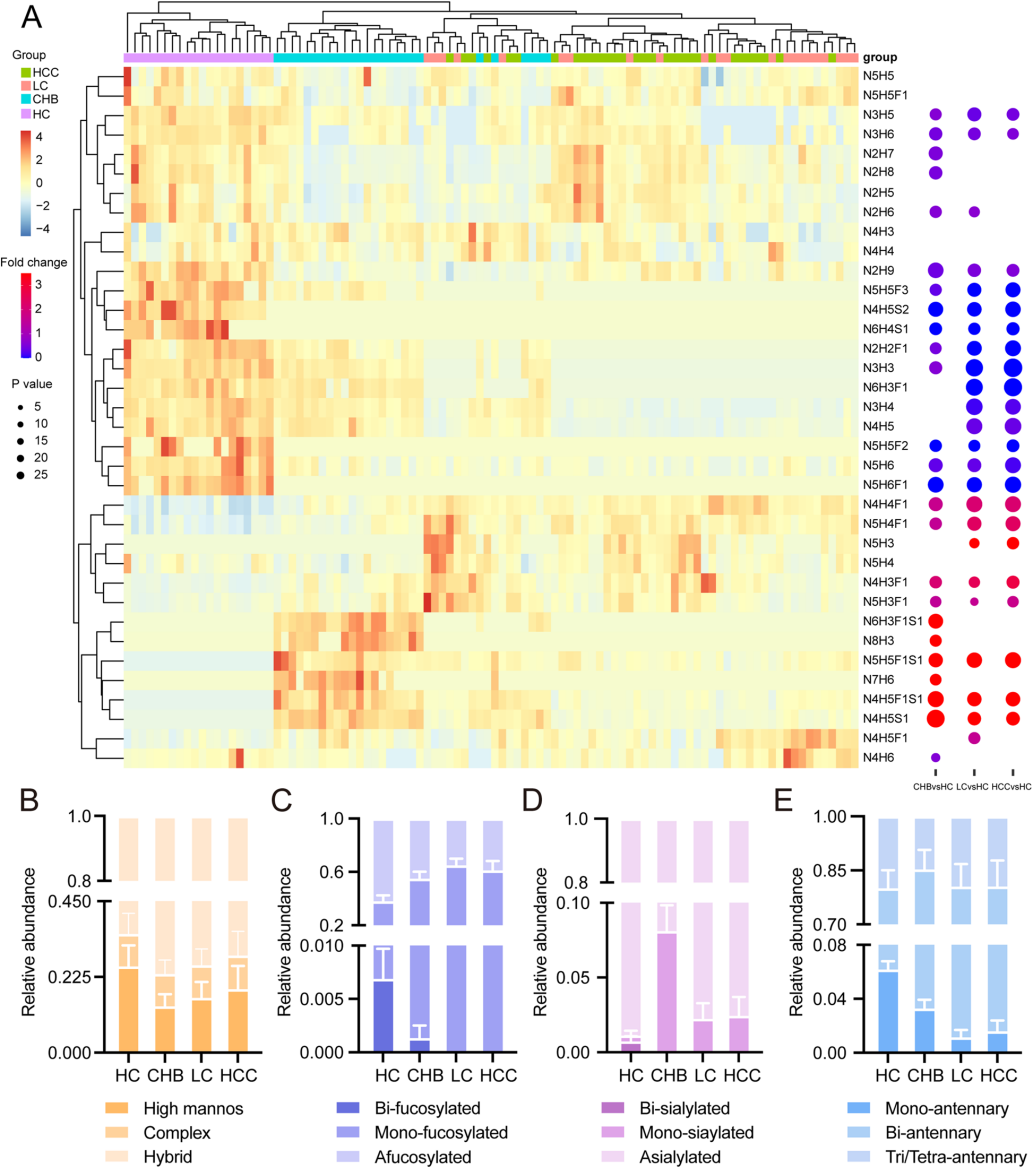
N-glycan levels in HC, CHB, LC and HBV-HCC serum samples. **A** Expression pattern and dysregulation of N-glycans identified in HC, CHB, LC and HBV-HCC. **B** Relative abundances of high mannose, complex and hybrid N-glycans. The relative abundance is calculated by adding the relative abundances of a given type of N-glycan. **C** Relative abundances of bi-, mono- and afucosylated N-glycans. **D** Relative abundances of bi-, mono- and asialylated N-glycans. **E** Relative abundances of N-glycans with mono-, bi- and tri/tetra-antennary structures

The expression pattern of identified N-glycans in HC, CHB, LC and HBV-HCC were exhibited in Fig. 3A. Hierarchical clustering revealed that LC/HBV-HCC were clearly separated from HC and CHB, however, LC/HBV-HCC could not be separated from each other. Furthermore, quantitative comparison analysis revealed that 25, 24, and 22 N-glycan structures were differentially expressed in CHB, LC and HBV-HCC versus HC. Of these N-glycans, 11 N-glycans were concertedly down-regulated, and 7 N-glycans were concertedly up-regulated in the progression of HBV-HCC. Notably, All up-regulated N-glycans were fucosylated (Fig. 3A; Table S5).

N-glycans were classified into three types, including high mannose, complex and hybrid. Relative abundance of hybrid and complex N-glycans were increased in CHB, LC and HBV-HCC versus HC, while which of high mannose N-glycans were decreased (Fig. 3B). Consistent with elevated FUTs expression at mRNA levels, relative abundance of total fucosylation were up-regulated, of which mono-fucosylation levels were increased, while bi-fucosylation levels were decreased (Fig. 3C). Other terminal modification sialylation levels were increased, followed by a decrease in the progression of HBV-HCC (Fig. 3D). Significantly higher levels of bi-antennary structures were found in CHB, LC and HBV-HCC versus HC, while mono-antennary structures was progressively reduced (Fig. 3E). In combination of transcriptomic and glycomic analysis revealed that fucosylation levels were up-regulated in the progression of HBV-HCC.

### Site-specific glycoproteomic profiling in HBV-HCC serum samples

**Fig. 4:**
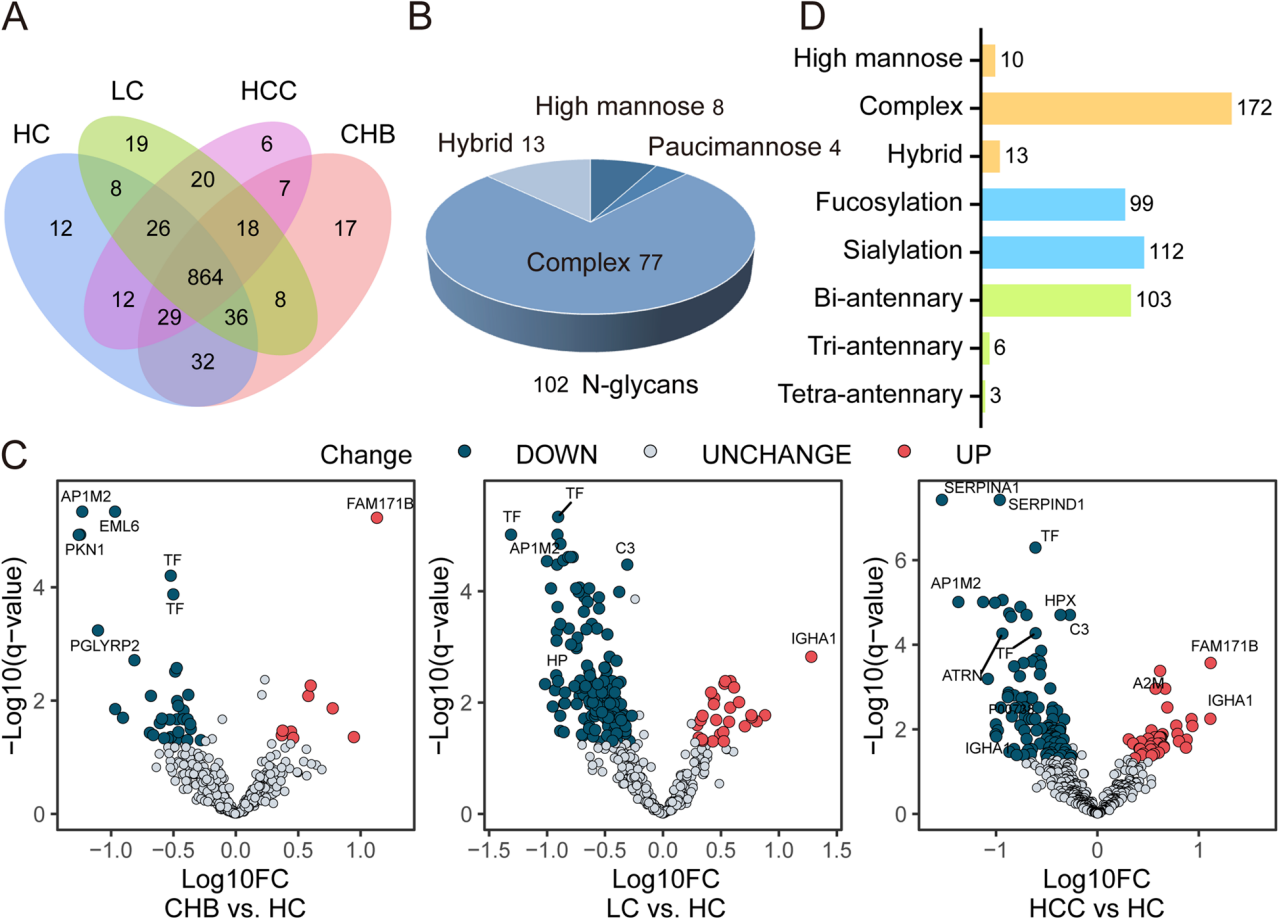
Quantitative glycoprotemic analysis of HC, CHB, LC and HBV-HCC serum samples. **A** Venn diagram of intact glycopeptides identified from HC, CHB, LC and HBV-HCC samples. **B** Distribution of glycan subtypes from intact glycopeptides identified from HC, CHB, LC and HBV-HCC samples. **C** Volcano plots of expression patterns of identified glycopeptides. Red dots: up-regulated glycopeptides. Green dots: down-regulated glycopeptides. The value q (-log10) is plotted against the log10 (FC: disease group vs. normal group). **D** Classification of differentially expressed intact glycopeptides based on their attached glycan structures. The numbers indicate the unique glycopeptides modified by the corresponding glycans

To decode the biological function of specific N-glycosylation especially fucosylation in the progression of HBV-HCC, intact glycoproteomic analysis of HC, CHB, LC and HBV-HCC serum samples (Table S2) were performed. A total of 1114 glycopeptides were identified (Fig. 4A; Table S6), of which, 1019, 1011, 999 and 982 glycopeptides, including 864 in common were found in HC, CHB, LC and HBV-HCC serum samples, respectively. The identified glycopeptides represented 129 glycosites were modified with 102 glycan structures. These N-glycans contained 8 high mannose, 13 hybrid, 77 complex and 4 paucimannose subtypes (Fig. 4B).

Quantitative comparison analysis revealed that 116, 197 and 192 glycopeptides were differentially expressed in HC compared to CHB, LC and HCC, respectively, using the cutoff of fold change > 1.5 or < 0.67 and *p* value < 0.05 (Fig. 4C). These 288 differentially expressed glycopeptides (DEGPeps) from 73 glycoproteins were mainly decorated with complex type N-glycans, of which sialylated N-glycans accounted for the largest proportion, followed by fucosylated N-glycans and bi-antennary structures (Fig. 4D).

**Fig. 5:**
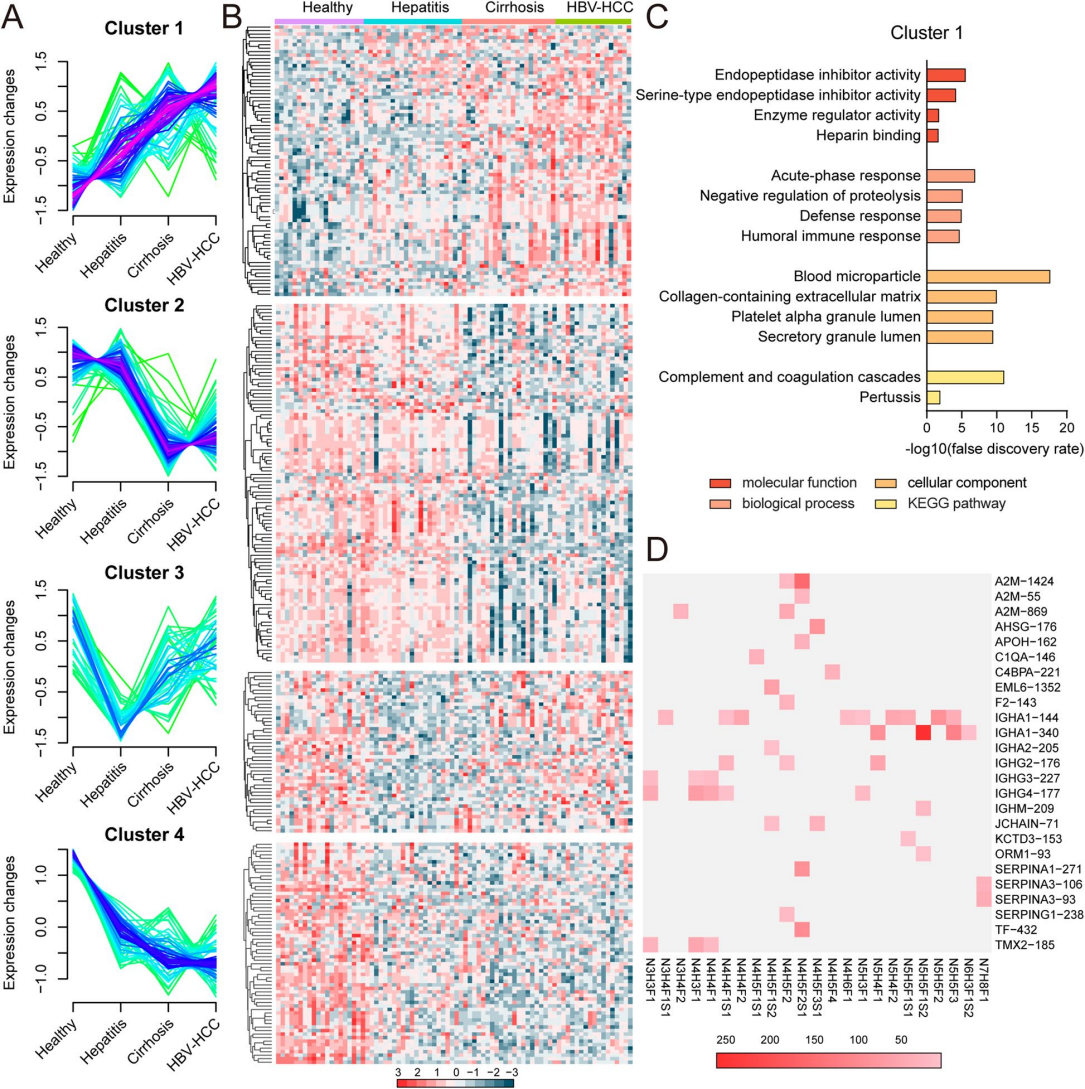
Hierarchical clustering analysis of differentially expressed glycopeptides identified in HC, CHB, LC and HBV-HCC. **A** Mfuzz clustering of differentially expressed glycopeptides. **B** Expression pattern of glycopeptides from cluster I, II, III and IV. **C** Functional enrichment analysis of differentially expressed glycopeptides from cluster I. **D** Heatmap of fucosylated N-glycans on intact glycopeptides from cluster I. PSMs of the intact glycopeptides, comprising of different glycans (bottom) and their glycosite locations in different glycoproteins (right) are exhibited in the heat map

To understand the association between glycoprotein trajectories and the progression of HBV-HCC, hierarchical clustering for all DEGPeps was performed, with which four cluster (I-IV) were generated (Fig. 5A&B; Table S7). Glycopeptides in “cluster I” were continually increased in the progression of HBV-HCC, encompassing 27 proteins. These DEGPeps were significantly enriched in endopeptidase inhibitor activity, acute-phase response, blood microparticle and complement and coagulation cascades by functional enrichment analysis (Fig. 5C). Glycopeptides in “cluster II” were significantly decreased, followed by an increase (39 proteins), and mainly involved in the peptidase regulator activity, complement activation, blood microparticle and complement and coagulation cascades. Glycopeptides in “cluster III” were slightly decreased, followed by an increase (25 proteins), and enriched in regulation of humoral immune response, blood microparticle and complement and coagulation cascades. Glycopeptides in “cluster IV” (31 proteins) were decreased and primarily connected with peptidase regulator activity, acute-phase response, blood microparticle, complement and coagulation cascades. (Fig. S2).

Taking into account the escalating malignancy of HBV-HCC progression, 49 glycopeptides with fucosylation attached in “cluster I” were screened out, which were consisted of 23 fucosylated N-glycans and 25 peptides from 21 protiens (Fig. 5D). These fucosylated N-glycans contained three to seven HexNAc, three to eight hexose, up to four fucose, and two sialic acid. Among 21 identified glycoproteins, 16 contained 1 glycosite, 3 contained 2 glycosites, and 1 contained 3 glycosites. Notably, among these glycopeptides, IgA1-340-N5H5F1S2 was identified with highest PSM score.

### Site-specific fucosylation on IgA1 and IgG2

**Fig. 6:**
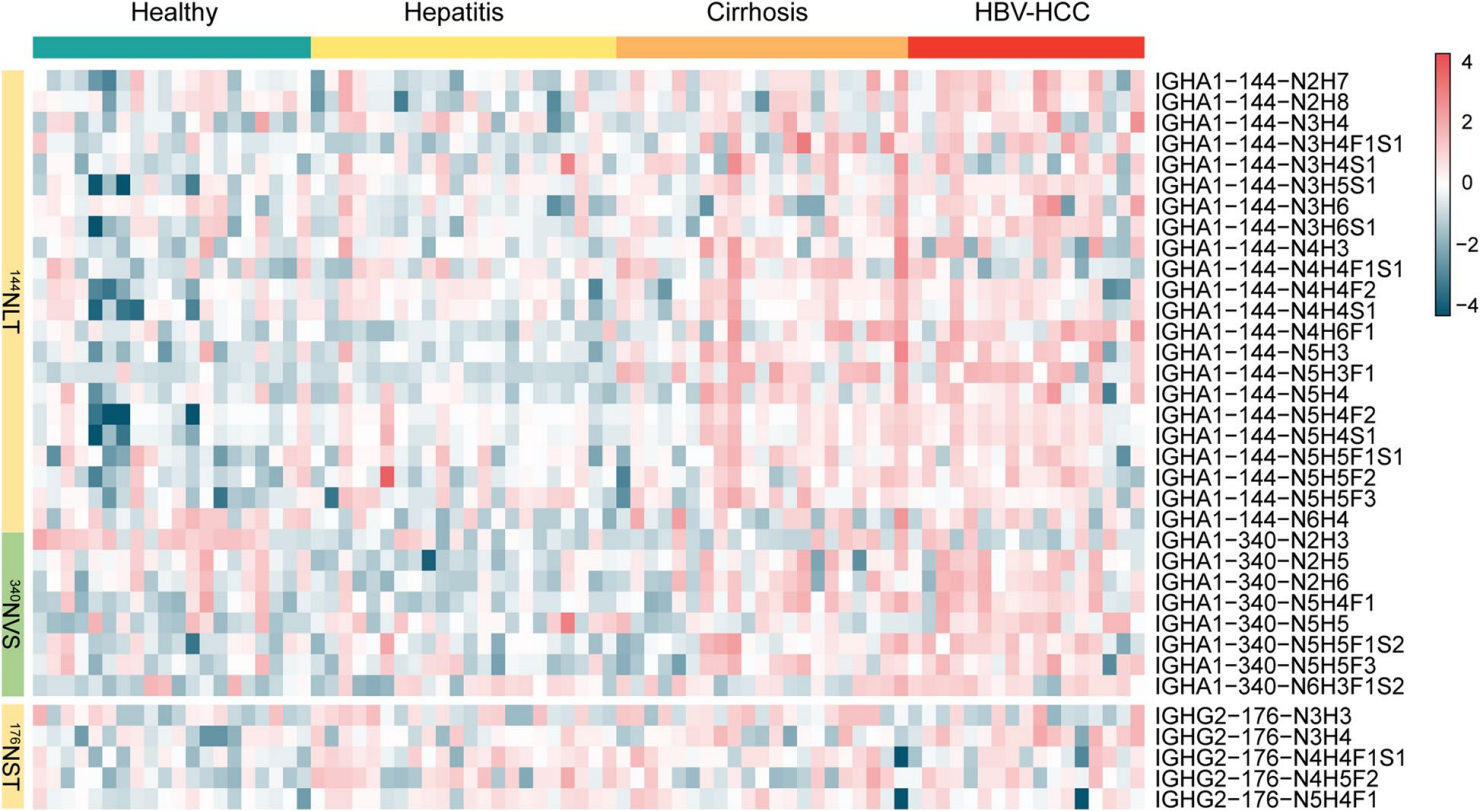
Site-specific glycan profiling of IGHA1 and IGHG2 identified in HC, CHB, LC and HBV-HCC. Heatmap showing all the identified glycans at the glycosite Asn-144, Asn-340 of IGHA1, and Asn-176 of IGHG2

We further mapped N-glycan structures on each glycosite of DEGPeps from IgA1 and IgG2 based on our glycoproteomics data. A total of 30 and 5 unique intact glycopeptides were identified from IgA1 and IgG2, which were comprised 3 glycosites (^144^N^#^LT and ^340^N^#^VS for IgA1, ^176^N^#^ST for IgG2) and 31 glycans (Fig. 6A). Among these glycopeptides, fucosylation was accounted for 46% of all glycans on glycopeptides, and the majority of fucosylated intact glycopeptides were up-regulated in the progression of HBV-HCC.

**Fig. 7:**
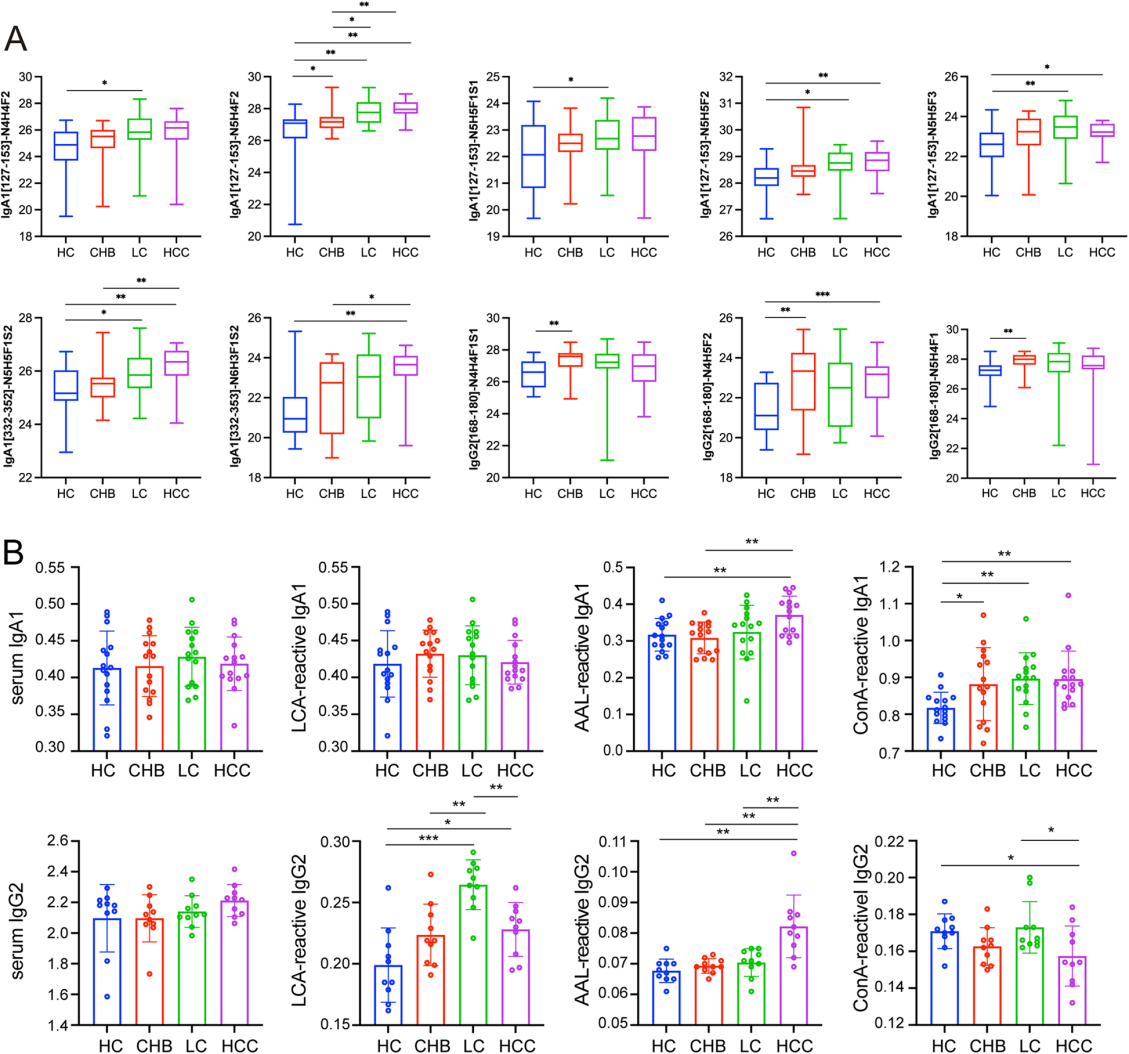
Differentially expressed intact glycopeptides of IGHA1 and IGHG2. **A** Box plots of 10 site-specific fucosylated glycopeptides from IgA1 and IgG2 showed a directionally concerted up-regulation in the HC-CHB-LC-HCC progression. **B** Relative expression of IgA1/ IgG2 and ConA-/ LCA-/ AAL-reactive IgA1/ IgG2 in the HC, CHB, LC and HBV-HCC serum determined by ELISA

Among these DEGPeps, 7 and 3 site-specific fucosylated glycopeptides from IgA1 and IgG2, showed a directionally concerted up-regulation in the HC-CHB-LC-HCC progression (Fig. 7A). To evaluate the diagnostic value of the aberrant fucosylated Igs in clinical practice, lectin-based ELISA assay with *Concanavalin A* (ConA), *Lens Culinaris* Agglutinin (LCA) and *Aleuria Aurantia* Lectin (AAL) was performed, which respectively recognize high mannose type N-glycans, α1,6-fucose, and α1,2/α1,3/α1,4/α1,6-fucose. The results revealed that total levels of IgG2 and IgA1 were not significantly changed, and different glycoform on Igs exhibited different expression patterns in the progression of HBV-HCC (Fig. 7B). AAL-reactive IgA1 and IgG2 were significantly elevated in HBV-HCC compared to HC, CHB and LC. LCA-reactive IgA1 was increased in HBV-HCC when compared to HC and decreased compared to CHB and LC, and LCA-reactive IgG2 was gradually increased and followed by a decrease in the progression of HBV-HCC. ConA-reactive IgA1 were increased in CHB, LC and HBV-HCC compared to HC, and ConA-reactive IgG2 showed lowest levels in HBV-HCC group. The different performance of these different glycoform on Igs indicated that glycosylation on Igs were specifically altered in the progression of HBV-HCC.

In summary, combined results of glycoproteomic and ELISA analysis revealed that IgA1 and IgG2 are highly fucosylated and AAL-reactive fucosylated IgA1 and IgG2 were up-regulated in HBV-HCC, implying their potential diagnostic value.

### Proteomic analysis of HBV-HCC serum samples

**Fig. 8:**
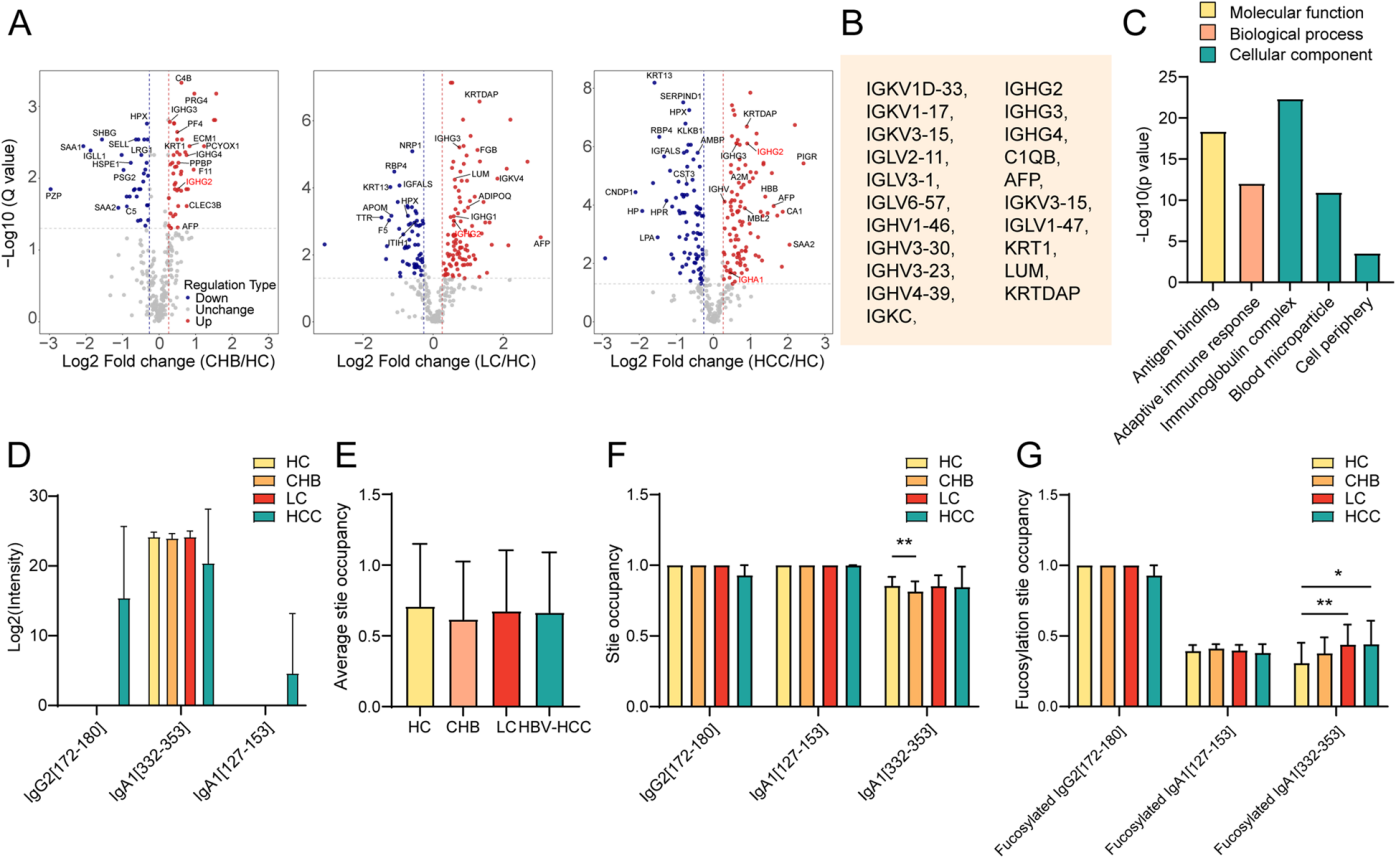
Glycosylation site occupancy. **A** Volcano plots of expression patterns of identified proteins. Red dots: up-regulated proteins. Green dots: down-regulated proteins. The value q (-log10) is plotted against the log2 (FC: disease group vs. normal group). **B** Lists of concertedly up-regulated proteins in patients with liver disease (CHB, LC, or HBV-HCC) compared to HC. **C** GO enrichment analysis of the concertedly up-regulated proteins in HBV-HCC. **D** Levels of peptides from IgA1 and IgG2, including IgA1[127–153], IgA1[332–353] and IgG2[168–180] in HC, CHB, LC and HBV-HCC. **E** Average glycosylation site occupancy in HC, CHB, LC and HBV-HCC. Glycosylation site occupancy was calculated by dividing the abundance of a given type of glycoform by the total corresponding peptide (glycopeptide and non-glycopeptide) abundance. **F** Glycosylation site occupancy of IgA1[127–153], IgA1[332–353] and IgG2[168–180]. **G** Fucosylation site occupancy of IgA1[127–153], IgA1[332–353] and IgG2[168–180]

To comprehend the potential of the glycoproteins analysis and conclusions, proteomics of these samples have been performed, revealing 78, 163, and 186 differentially expressed proteins in HC compared to CHB, LC and HCC, respectively (Fig. 8A, Table S8). Among these differentially expressed proteins, 21 proteins, including AFP and diverse Igs, were up-regulated (*p* < 0.05) in patients with liver disease (CHB, LC, or HBV-HCC) compared to HC (Fig. 8B), that were mainly enriched in the cellular components of immunoglobulin complex, blood microparticle and cell periphery, the biological process of adaptive immune response, and molecular function of antigen binding (Fig. 8C). Notably, IgA1 did not show significant differences, while IgG2 was significantly up-regulated in patients with liver disease compared to HC (Fig. 8B). Peptides corresponding to the differentially expressed glycopeptides of Igs were investigated. The non-glycosylated peptides from IgA1 (amino acids 332–353; termed as IgA1[332–353]) did not show significant differences in the progression of HBV-HCC, while IgA1[127–153] and IgG2[172–180] were only detected in HBV-HCC (Fig. 8D), suggesting that fucosylated peptides from IgA1 and IgG2, especially IgA1[332–353], were specially up-regulated in the progression of HBV-HCC.

Furthermore, we correlated the abundance of glycopeptides with total corresponding peptides levels to determine the glycosylation site occupancy (Table S9). We found that the average site occupancy did not significantly changed (Fig. 8E). The glycosylation site occupancy of IgA1[127–153] and IgG2[172–180] exhibited no significant changes in the progression of HBV-HCC, and which of IgA1[332–353] was decreased in CHB compared to HC (Fig. 8F). Specifically, N-glycans on IgG2[172–180] were mainly fucosylated, and fucosylation site occupancy of IgA1[332–353] was elevated in LC and HCC compared to HC (Fig. 8G).

Collectively, When the IgA1 remains unchanged at protein levels, the fucosylation site occupancy of IgA1[332–353] increases, leading to a rise in IgA1[332–352]-N5H5F1S2 or -N6H3F1S2 and consequently resulting in elevated levels of AAL-reactive IgA1 in HBV-HCC. When fucosylation site occupancy of IgG2[168–180] remains unchanged, the IgG2 exhibited a upregulation at protein levels, contributing to a rise in IgG2[168–180]-N4H5F2 and consequently leading to a elevated level of LCA- and AAL-reactive IgG2 in HBV-HCC.

## Discussion

An alteration in glycosylation have long been observed with the progression of HBV-HCC [36, 37]. Our study comprehensively characterizes the transcriptomics, glycomics and glycoproteomics landscapes of HC, CHB, LC and HBV-HCC, which allows us to interrogate underlying mechanisms in HBV-HCC progression. Our transcriptomic analysis revealed that several FUTs responsible for the N-glycan fucosylation were dysregulated in HBV-HCC (Fig. 1F). FUT1 catalyzes the addition of fucose to a terminal galactose in an α1,2-linkage, while FUT4, 6, 7, 9 catalyze the formation of α1,3-fucosylation on terminal GlcNAc, and FUT8 catalyze the core-α1,6-fucosylation on the innermost GlcNAc of core pentasaccharide. Consistently, our glycomic analysis revealed that most of concertedly up-regulated N-glycans in the progression of HBV-HCC were annotated to be fucosylated (Fig. 3C), in line with the up-regulation of FUT1, 4, 8. Recent studies also observed an elevated serum core-fucosylation by HPLC [38], and an elevated saliva fucosylation by saliva microarrays with *Lotus Tetragonolobus* Lectin [39] in the progression of human HBV-HCC. Moreover, a serum-associated fucosylated glycoprotein (< 10 kD) fingerprint was established to assist the diagnosis of HBV-HCC by using MALDI-TOF–MS, which could distinguish HBV-HCC from healthy subjects, patients with HBV and HBV-associated cirrhosis [40].

Furthermore, we found that most of concertedly up-regulated N-glycans were decorated with mono-fucosylated bi-/tri-antennary. Specifically, the up-regulation of the core-fucosylated monogalactose biantennary glycan (N4H4F1) is in agreement with the previously reported study [34]. In situ glycan imaging also revealed that fucosylation mostly on tetra-antennary and to a lesser extent on bi-antennary and tri-antennary glycans were elevated in HCC [24] in line with the elevated fucosylated tri-antennary N-glycans (N5H3F1, N5H4F1, N5H5F1) in our glycomic analysis. Xue-En Liu et al. found that a branch α1,3-fucosylated tri-antennary N-glycan (NA3Fb) was elevated in patients with HBV-HCC compared to who with HBV-fibrosis or cirrhosis or healthy subjects by using a DNA sequencer [41], furthermore, the log ratio of NA3Fb/NG1A2F was elevated in HBV-HCC, which was promising as N-glycan markers [42]. While, we found that levels of N5H6F1 N-glycan (the same component as NA3Fb) were reduced in HBV-HCC. This discrepancy might arise from inefficient distinction between N-glycan isoforms of MALDI-TOF–MS.

Advances in glycomics, genomics and proteomics platforms over the last decade have discovered numerous novel biomarkers and have improved the diagnosis of HBV-HCC. In recent years, the glycoproteome mapping provides innovative insights into the both peptides and glycosylation status in diseases and contributes to biomarker discovery. As the predominant constituent in immune molecule, Ig glycosylation especially fucosylation variations were found to be closely associated with the development of HBV-HCC using glycoproteomics [43]. Core-fucosylated serum IgG was significantly increased in HBV-HCC compared to HBV-related cirrhosis, HBV carriers and healthy subjects, and have general diagnostic performance than AFP in the training set and validation cohorts [44]. Similar research revealed that TPLTAN^205^ITK (H5N5S1F1) and (H5N4S2F1) of IgA2 were significantly elevated in serum from patients with HBV infection and even higher in HBV cirrhosis in comparison with healthy donor [45]. Consistently, in the present study, 30 N-glycan structures on 2 glycosites of IgA1 and 5 N-glycans on 1 glycosite of IgG2 were identified. Specifically, differentially expressed IgA1 with fucosylated glycan structures (IgA1-144-N4H4F2/N5H4F2/N5H5F1S1/N5H5F2/N5H5F3 and IgA1-340-N5H5F1S2/N5H5F3/N6H3F1S2) and IgG2 with fucosylated glycan structures (IgG2-176-N4H3F1/N4H4F1/N5H3F1) were elevated in the progression of HBV-HCC determined by glycoproteomics.

Since each fucosylated glycoform can be recognized by a distinct lectin, a lectin-based ELISA was used to quantify the fucosylated Igs, which is more appropriate for the clinical practice. The present study revealed that the elevated AAL-reactive IgA1 and IgG2 in HBV-HCC might distinguish HBV-HCC from HC, CHB and LC. Similarly, the reduced LCA-reactive IgA1 in HBV-HCC might distinguish HBV-HCC from other groups, while the elevated LCA-reactive IgG2 in CHB, LC and HBV-HCC might distinguish liver diseases from HC, and even distinguish LC from other groups. These results indicated that LCA- and AAL-reactive Igs showed a diagnostic potential in distinguishing HBV-HCC from HC, CHB and LC.

By integrating omics and ELISA data, IgG2 levels were continuously elevated in patients with liver disease (CHB, LC, or HBV-HCC) compared to HC determined by proteomics (Fig. 8B), and which was slightly but not significantly increased in liver disease determined by ELISA (Fig. 7B). Notably, AAL- and LCA-reactive IgG2 were significantly elevated in HBV-HCC compared to HC (Fig. 7B), which might attribute to the changes in IgG2-176-HexNAc(4)Hex(5)Fuc(2) (Fig. 7A). Moreover, LCA-reactive IgG2 were up-regulated in CHB compared to HC (Fig. 7B), which might result from the variants of fucosylated glycopeptides including IgG-176-HexNAc(4)Hex(4)Fuc(1)NeuAc(1), HexNAc(4)Hex(5)Fuc(2), and HexNAc(5)Hex(4)Fuc(1) (Fig. 7B). These results reveal a specific expression pattern of fucosylated IgGs, distinct from non-glycosylated IgGs, indicating their potential diagnostic value in HCC.

In addition to fucosylation, sialylation plays an essential role in cell–cell recognition, cell adhesion, antigenicity, protein targeting and invasion [46–48], is closely associated with HCC [49]. In the present study, sialylation levels were continuously elevated in CHB, LC and HCC compared to HC (Fig. 3D), consistent with the increased sialylation levels in serum samples of HCC patients [50]. Moreover, sialylated glycoproteins were documented to be dysregulated in the progression of HCC, including AFP [51] and paraoxonase 1 [52]. In the present study, 39 sialylated glycopeptides were continuously increased in HCC progression (Fig. S3), which were consisted of 26 sialylated N-glycans and 16 peptides from 23 glycoproteins. Among these glycopeptides, SERPINA1-271-HexNAc(4)Hex(5)NeuAc(1) was identified with highest PSM score. Aligning with this change, sialylated SERPINA1 were up-regulated in early-stage oropharyngeal squamous cell carcinoma [53] and in metastatic HCC compared to non-metastatic HCC group [54], suggesting that sialylated SERPINA1 was closely associated with HCC.

Therefore, we speculate that changes in serum IgG and IgA glycosylation may closely correlate to the progression of HCC, and act as complementary techniques to improve diagnosis.

### Supplementary Information

**Additional file 1:** **Fig. S1.** PPI network of other DEGGs. The PPI network of DEGGs performed by the STRING database and cytoscape tools. **Fig. S2.** Functional enrichment analysis of glycopeptides from cluster II (A), III (B) and IV (C). **Fig. S3.** Heatmap of sialylated N-glycans on intact glycopeptides from cluster I. PSMs of the intact glycopeptides, comprising of different glycans (bottom) and their glycosite locations in different glycoproteins (right) are exhibited in the heat map.

**Additional file 2: Table 1.** Characteristics of 100 participants clincal data of glycomics. **Table 2.** Characteristics of 80 of 100 participants clincal data of glycoproteomics. **Table 3.** Primers of glycan-related genes. **Table 4.** Differentially expressed glyco-genes in GSE135631 and GSE94660. **Table 5.** Identified N-glycans in HC, CHB, LC and HCC serum samples. **Table 6.** Identified glycopeptides in HC, CHB, LC and HCC serum samples. **Table 7.** Differentially expressed intact glycopeptides from cluster I-IV. **Table 8.** Differentially expressed proteins in CHB, LC and HBV-HCC compared HC. **Table 9.** Glycosylation site occupancy of glycopeptides in HC, CHB, LC and HBV-HCC.

## Data Availability

Raw data has been deposited into a jPOSTrepo repository [55] The accession link was https://repository.jpostdb.org/preview/2022.

## Abbreviation

HCC: Hepatocellular carcinoma
HBV: Hepatitis B virus
GEO: Gene Expression Omnibus
FUTs: Fucosyltransferases
HBV-HCC: HBV-associated HCC
AFP: Alpha-fetoprotein
β1,6-GlcNAc: β1,6-N-acetylglucosamine
TF: Transferrin
A1AT: Alpha-1-anti-trypsin
CHB: Chronic hepatitis B
LC: Liver cirrhosis
HC: Healthy controls
NCBI: National Center for Biotechnology Information
RT-qPCR: Quantitative reverse transcription PCR
DTT: Dithiothreitol
IAM: Iodoacetamide
SPE: Solid phase extraction
DHB: 2,5-Dihydroxybenzoic acid
LCA: Lens Culinaries Agglutinin
SD: Standard deviation
DEGGs: Differentially expressed glyco-genes
PLS-DA: Partial Least Squares Discrimination Analysis
PPI: Protein–protein interaction
DEGPeps: Differentially expressed glycopeptides
