# Supplementary figures and images for: Integrating transcriptomics, glycomics and glycoproteomics to characterize hepatitis B virus-associated hepatocellular carcinoma
Source: Cell Commun Signal. 2024 Apr 1;22:200. doi: 10.1186/s12964-024-01569-y (PMC10983713; doi:10.1186/s12964-024-01569-y)

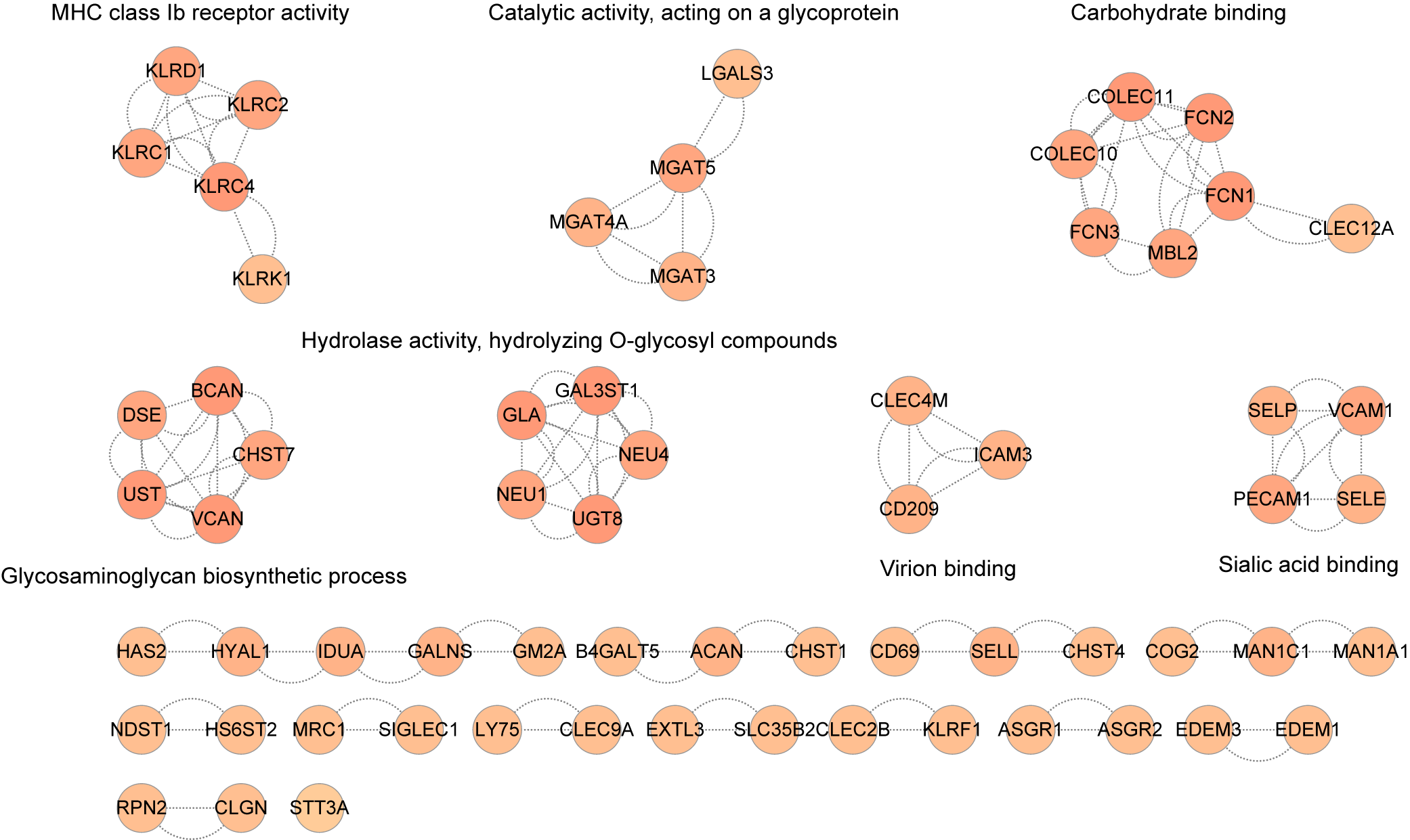

Supplement: Supplementary file 1 — Additional file 1: Fig. S1. PPI network of other DEGGs. The PPI network of DEGGs performed by the STRING database and cytoscape tools. Fig. S2. Functional enrichment analysis of glycopeptides from cluster II (A), III (B) and IV (C). Fig. S3. Heatmap of sialylated N-glycans on intact glycopeptides from cluster I. PSMs of the intact glycopeptides, comprising of different glycans (bottom) and their glycosite locations in different glycoproteins (right) are exhibited in the heat map. [file 12964_2024_1569_MOESM1_ESM.zip › Figure S1.tif]

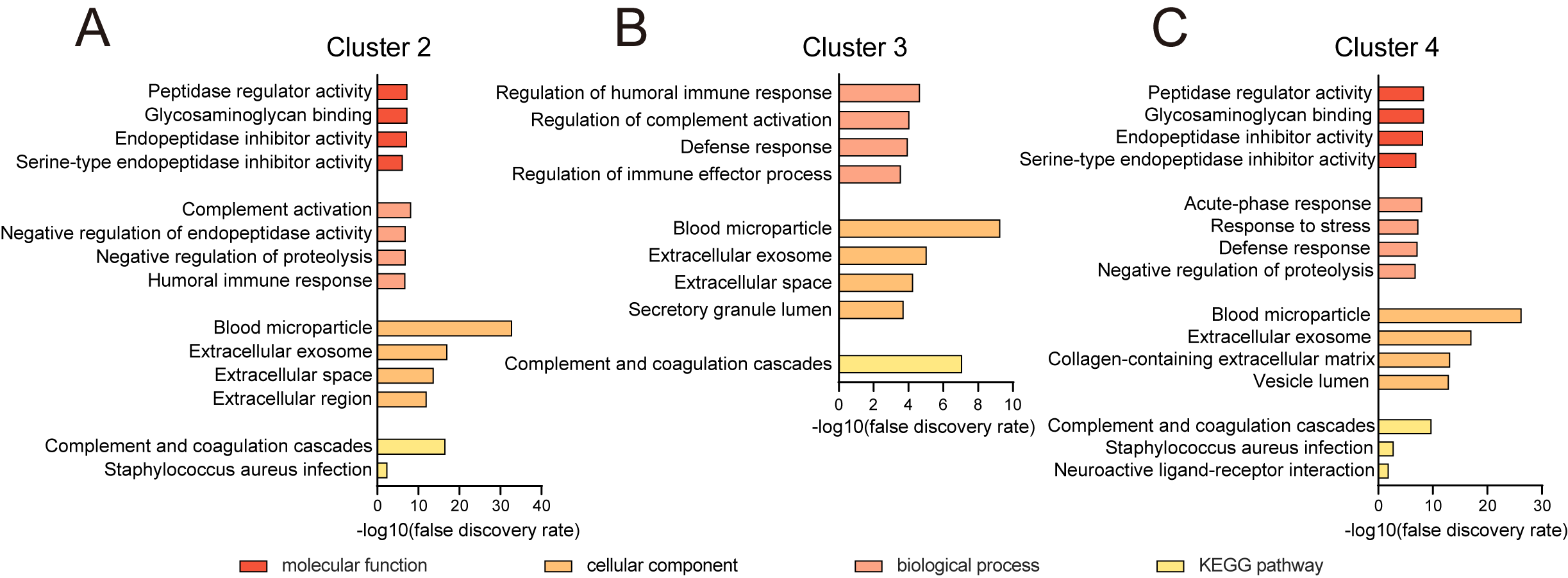

Supplement: Supplementary file 1 — Additional file 1: Fig. S1. PPI network of other DEGGs. The PPI network of DEGGs performed by the STRING database and cytoscape tools. Fig. S2. Functional enrichment analysis of glycopeptides from cluster II (A), III (B) and IV (C). Fig. S3. Heatmap of sialylated N-glycans on intact glycopeptides from cluster I. PSMs of the intact glycopeptides, comprising of different glycans (bottom) and their glycosite locations in different glycoproteins (right) are exhibited in the heat map. [file 12964_2024_1569_MOESM1_ESM.zip › Figure S2.tif]

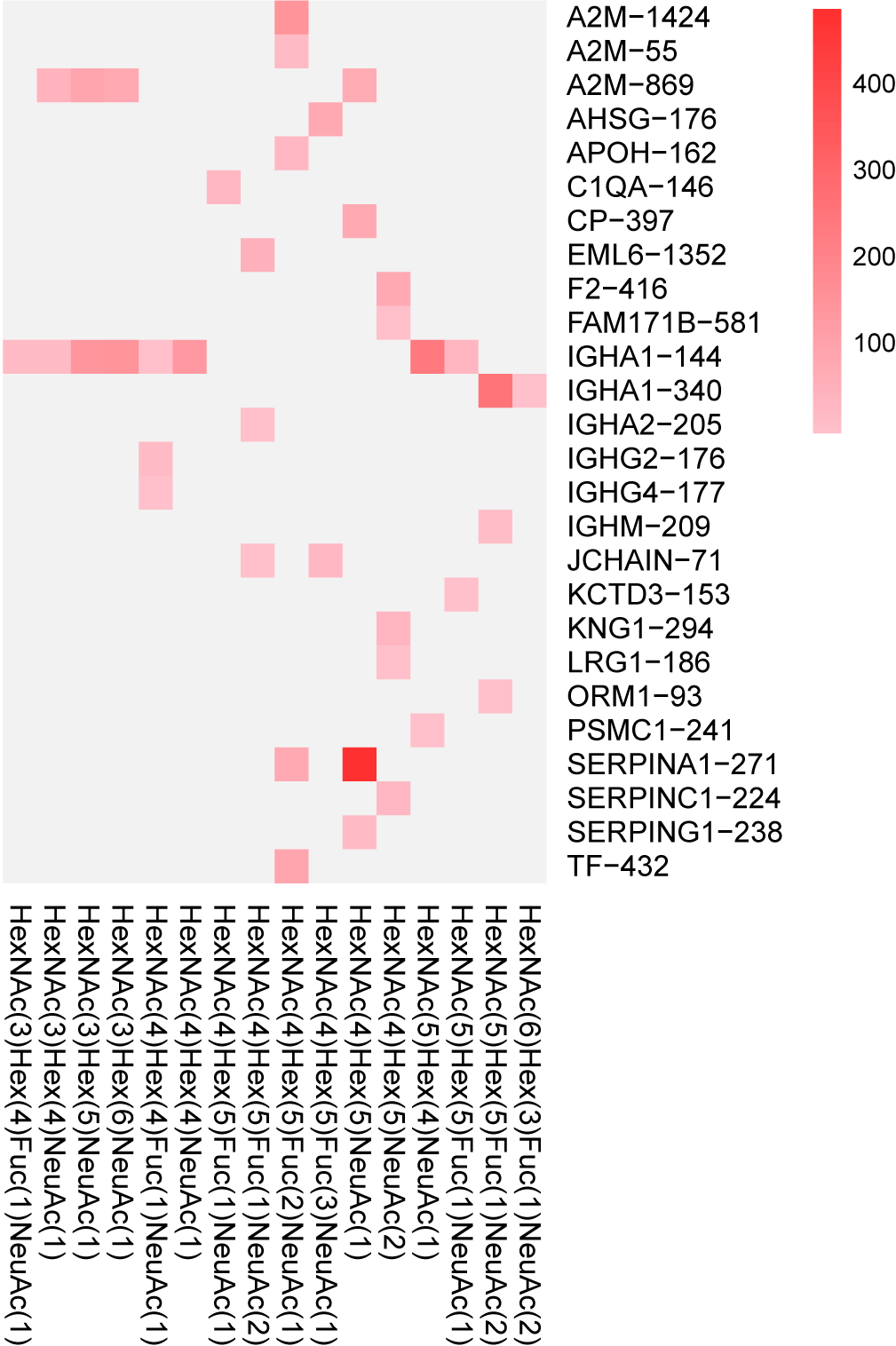

Supplement: Supplementary file 1 — Additional file 1: Fig. S1. PPI network of other DEGGs. The PPI network of DEGGs performed by the STRING database and cytoscape tools. Fig. S2. Functional enrichment analysis of glycopeptides from cluster II (A), III (B) and IV (C). Fig. S3. Heatmap of sialylated N-glycans on intact glycopeptides from cluster I. PSMs of the intact glycopeptides, comprising of different glycans (bottom) and their glycosite locations in different glycoproteins (right) are exhibited in the heat map. [file 12964_2024_1569_MOESM1_ESM.zip › Figure S3.tif]
