# Supplementary material for: Integrating transcriptomics, glycomics and glycoproteomics to characterize hepatitis B virus-associated hepatocellular carcinoma
Source: Cell Commun Signal. 2024 Apr 1;22:200. doi: 10.1186/s12964-024-01569-y (PMC10983713; doi:10.1186/s12964-024-01569-y)
Supplement: Supplementary file 2 — Additional file 2: Table 1. Characteristics of 100 participants clincal data of glycomics. Table 2. Characteristics of 80 of 100 participants clincal data of glycoproteomics. Table 3. Primers of glycan-related genes. Table 4. Differentially expressed glyco-genes in GSE135631 and GSE94660. Table 5. Identified N-glycans in HC, CHB, LC and HCC serum samples. Table 6. Identified glycopeptides in HC, CHB, LC and HCC serum samples. Table 7. Differentially expressed intact glycopeptides from cluster I-IV. Table 8. Differentially expressed proteins in CHB, LC and HBV-HCC compared HC. Table 9. Glycosylation site occupancy of glycopeptides in HC, CHB, LC and HBV-HCC. [file 12964_2024_1569_MOESM2_ESM.zip › 12964_2024_1569_MOESM2_ESM/Table S1&2.docx]

**Supporting information**

**Table 1** Characteristics of 100 participants clincal data of glycomics.

| Characteristics | HC (n=20) | CHB (n=27) | LC (n=22) | HCC (n=31) |
| --- | --- | --- | --- | --- |
| Gender |  |  |  |  |
| Male | 8 | 16 | 16 | 19 |
| Female | 12 | 11 | 6 | 12 |
| Age (years) |  |  |  |  |
| Mean±SD | 50.45±20.10 | 44.70±15.08 | 54.14±14.32 | 56.06±10.36 |
| HbsAg |  |  |  |  |
| Yes | / | 26 | 19 | 22 |
| No | / | 1 | 3 | 9 |
| AFP (ng/mL) |  |  |  |  |
| <2000.00 | 5.24±8.94 | 44.95±241.62 | 98.45±274.86 | 51.76±109.19 |
| >2000.00 | / | / | >2000.00 (2) | >2000.00 (6) |

**Table 2** Characteristics of 80 participants clincal data of glycoproteomics.

| Characteristics | HC (n=20) | CHB (n=22) | LC (n=21) | HCC (n=17) |
| --- | --- | --- | --- | --- |
| Gender |  |  |  |  |
| Male | 8 | 13 | 15 | 10 |
| Female | 12 | 9 | 6 | 7 |
| Age (years) |  |  |  |  |
| Mean±SD | 50.45±20.10 | 46.50±15.19 | 54.24±14.66 | 55.82±9.52 |
| HbsAg |  |  |  |  |
| Yes | / | 21 | 18 | 11 |
| No | / | 1 | 3 | 6 |
| AFP (ng/mL) |  |  |  |  |
| <2000.00 | 5.24±8.94 | 2.80±1.28 | 103.45±281.46 | 42.87±80.44 |
| >2000.00 | / | / | >2000.00 (2) | >2000.00 (3) |
